# Phytochemical and Ethnopharmacological Perspectives of *Ehretia laevis*

**DOI:** 10.3390/molecules26123489

**Published:** 2021-06-08

**Authors:** Pooja Sharma, Richa Shri, Fidele Ntie-Kang, Suresh Kumar

**Affiliations:** 1 Department of Pharmaceutical Sciences and Drug Research, Punjabi University, Patiala 147002, India or pooja.sharma2007@yahoo.co.in (P.S.); rshri587@hotmail.com (R.S.); 2 Sri Sai College of Pharmacy, Manawala, Amritsar 143001, India; 3 Department of Chemistry, Faculty of Science, University of Buea, Buea P.O. Box 63, Cameroon; 4 Institute for Pharmacy, Martin-Luther-Universität Halle-Wittenberg, Kurt-Mothes-Str. 3, 06120 Halle, Germany

**Keywords:** *Ehretia laevis*, pharmacological activities, phytochemistry, traditional use

## Abstract

*Ehretia laevis* Roxb. (Boraginaceae) has been extensively used as a traditional remedy for the treatment of a diverse range of ailments related to the respiratory system, the gastrointestinal tract, the reproductive system, and against several infections. This review critically assesses and documents, for the first time, the fragmented information on *E. laevis*, including its botanical description, folklore uses, bioactive phyto metabolites and pharmacological activities. The goal is to explore this plant therapeutically. Ethnomedicinal surveys reveal that *E. laevis* has been used by tribal communities in Asian countries for the treatment of various disorders. Quantitative and qualitative phytochemical investigations of *E. laevis* showed the presence of important phytoconstituents such as pentacyclic triterpenoids, phenolic acids, flavonoids, fatty acids, steroids, alkaloids, aliphatic alcohols, hydrocarbons, amino acids, carbohydrates, vitamins and minerals. Fresh plant parts, crude extracts, fractions and isolated compounds have been reported to exhibit broad spectrum of therapeutic activities viz., antioxidant, antiarthritic, antidiabetic, anti-inflammatory, antiulcer, antidiarrheal, antidysenteric, wound healing and anti-infective activities. *E. laevis* is shown to be an excellent potential source of drugs for the mitigation of jaundice, asthma, dysentery, ulcers, diarrhea, ringworm, eczema, diabetes, fissure, syphilis, cuts and wounds, inflammation, liver problems, venereal and infectious disorders. Although few investigations authenticated its traditional uses but employed uncharacterized crude extracts of the plant, the major concerns raised are reproducibility of therapeutic efficacy and safety of plant material. The outcomes of limited pharmacological screening and reported bioactive compounds of *E. laevis* suggest that there is an urgent need for in-depth pharmacological investigations of the plant.

## 1. Introduction

*Ehretia laevis* is a rapidly growing medium sized tree of the Boraginaceae. The genus *Ehretia* contains more than 150 species [1]. The plant is primarily distributed throughout tropical and subtropical regions of Asia, Africa and Australia [2]. *E. laevis* is the most popular member of its genus and is commonly known by more than 120 names in diverse languages (see Supplementary Materials Table S1). In India the plant is mainly found in the Northern parts of the country (e.g., in Bengal, Maharashtra and Rajasthan) [3,4]. The plant has also been documented in the traditional system of medicine (e.g., Ayurveda and Siddha). This is due to its extensive uses to treat respiratory system diseases (e.g., asthma and cough), gastrointestinal tract infections (e.g., jaundice, diarrhea, ulcers, dysentery, liver diseases), endocrine system diseases (e.g., diabetes mellitus), microbial infections (e.g., diphtheria, scabies, ringworm, gonorrhea, syphilis and venereal diseases) [5]. Some pharmacological screening studies have been performed to authenticate its traditional claims.

Moreover, several bioactive metabolites such as pentacyclic triterpenoids, phenolics, flavonoids, tannins, fatty acids, vitamins, minerals, amino acids and carbohydrates have been isolated from it’s crude extracts [6,7,8,9]. Several research groups have shown the presence of alkaloids, glycosides, flavonoids, phenolic acids, tannins, saponins, proteins and carbohydrates in the plant [10]. The pharmacological activities and outcomes of these investigations have not been able to sufficiently authenticate the possible mechanisms of action of these molecules. Therefore, the plant should be subjected to mechanistic studies at the molecular levels.

Additionally, only a few toxicity studies have been published yet, including acute oral toxicity studies on the crude extracts of *E. laevis*, confirming that the plant is safe through oral route at the dose of 2000 mg/kg [8]. Until now, not even a single study has been performed in detail for clinical trials. So, detailed toxicity, preclinical and clinical trials on the plant should be carried out in order to ensure its clinical efficacy and safety in humans.

However, no review is currently available which covers all aspects of *E. laevis*. The present review article covers botanical distribution, conventional uses, phytomolecules, toxicology and scientifically verified pharmacological activities of the plant and its metabolites, this justifying this review.

## 2. Materials and Methods

All pertinent information about the botanical description, conventional uses, phytomolecules and pharmacological activities of *E. laevis* were collected from available literature. The electronic databases employed for the assortment of relevant information include Scopus (accessed on 1 May 2021), NISCAIR (accessed on 1 May 2021), Scifinder (accessed on 1 May 2021), PubMed (accessed on 1 May 2021), Springer Link (accessed on 1 May 2021), Science Direct (accessed on 1 May 2021), Google Scholar (accessed on 1 May 2021), Web of Science (accessed on 1 May 2021) and exhaustive library search. The chemical structures of compounds were drawn using ChemDraw Ultra 8.0 software (PerkinElmer, Waltham, MA, USA). Pubchem (accessed on 1 May 2021) and ChemSpider (accessed on 1 May 2021) database has been used to check the IUPAC names of the isolated phytoconstituents.

### 2.1. Botanical Description

**Figure 1:**
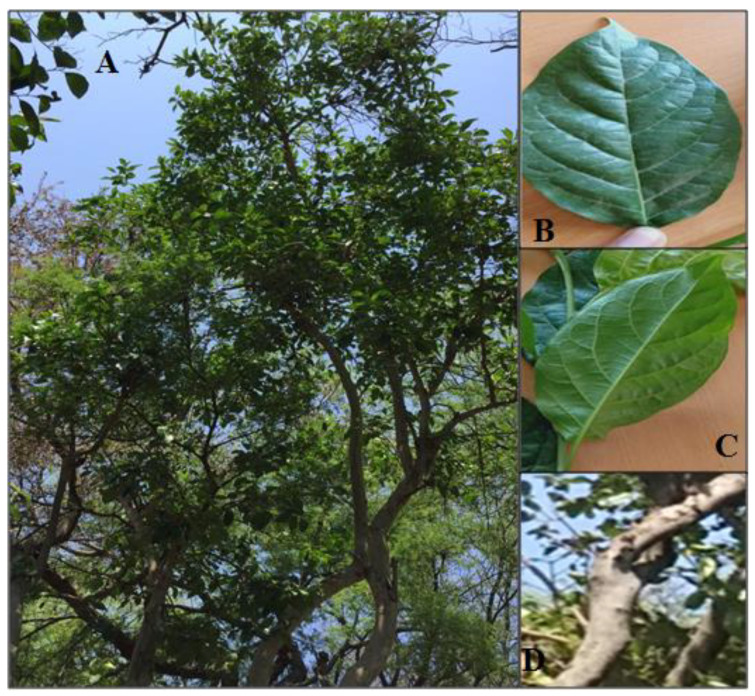
*E. laevis* tree (**A**); dark green leaf (**B**); leaf venation (**C**); stem-bark (**D**).

*Ehretia laevis* is a medium sized tree reaching up to the height of 12 m (Figure 1). Its dropping branches bear dark green colored leaves with varied size 2–7.8 cm in length and 1.2 cm to 3.8 cm in width. The shape of leaves is obtuse; with 5 to 7 lateral veins on each side of the mid rib with a slender 2–3 cm long petiole. The bark of the plant is irregular and light grey. The flowers are white, with round orange fruits when ripe or mature [11,12].

### 2.2. Geographical Distribution

*E. laevis* is mainly cultivated in India, China, Pakistan, Sri Lanka, Africa, Bhutan, Nepal, Burma, Vietnam and Australia. The plant is mainly located in hilly forests and on hilly slopes [2,13].

## 3. Local and Contemporary Uses of *E. laevis*

Several parts of *E. laevis* have been widely used for the traditional treatment of several disorders, including its leaves, barks, stems, seeds and fruits in different forms alone and sometimes also in combination with other herbs [5,8]. In Ayurveda *E. laevis* had been documented for its use in the treatment of asthma, diphtheria, cuts and wounds, astringent, fractures, demulcent, diarrhea, dysentery, toothache, cough, syphilis, gonorrhea cachexia, and venereal diseases [5,14].

It was found that various tribal communities used paste prepared from the leaves of *E. laevis* for the relief of acute and chronic inflammatory disorders [15]. Similarly, the folklore practitioners from Dhule district of Maharashtra recommended the use of the stems of *E. laevis* to treat ulcers and gum problems [16]. The leaf paste is also used for the management of fissures in the Wardha district of Maharashtra [17]. The Garasians tribes of Rajasthan use a decoction prepared from the plant bark for the relief of asthma, cough, cold and delivery pain [18]. In addition to this, an ethnobotanical survey conducted among elderly persons and traditional healers of tribal communities in the Jalpaiguri district of West Bengal revealed that the paste prepared from the bark of this plant relieves pains, especially in the lower limbs [19].

Another survey conducted on Gujjar tribes of Uttarakhand reported that *E. laevis* had been greatly used for the treatment of liver diseases, e.g., jaundice. A paste of soaked seeds of *E. laevis* was prepared, mixed with powder of *Amomum subulatum* and given thrice a day with milk by Nomadic people for the treatment of liver diseases [20]. Furthermore, an ethnobotanical survey has been conducted in the Tharu community of district Udham Singh Nagar, Uttarakhand, India. It was found that local villagers and health practitioners used the paste prepared from the seeds of *E. laevis* for the healing of skin diseases [21]. Leaves, stems, barks and fruits of *E. laevis* are also used in the manufacturing of dyes, cosmetics and wines [2,22]. All the local and contemporary uses of *E. laevis* mentioned in Ayurveda, Siddha and ethnobotanical surveys reports have been summarized in Table 1.

## 4. Phytochemistry

Ethnobotanical studies established that barks, leaves and fruits of *E. laevis* are potential sources of phytoconstituents. Phytochemical investigations had led to the extraction and isolation of secondary metabolites along with primary metabolites from petroleum ether, chloroform and methanolic extracts of its barks and leaves. These are pentacyclic triterpenoids, flavonoids, alkaloids, tannins, phenolic components, phenolic acids, hydrocarbons, aliphatic alcohols, fatty acids, ascorbic acid, amino acids, carbohydrates, benzoquinones, vitamins and minerals [6,7,8,9,34,38,39,40,41].

### 4.1. Pentacyclic Triterpenoids and Phytosterol

**Figure 2:**
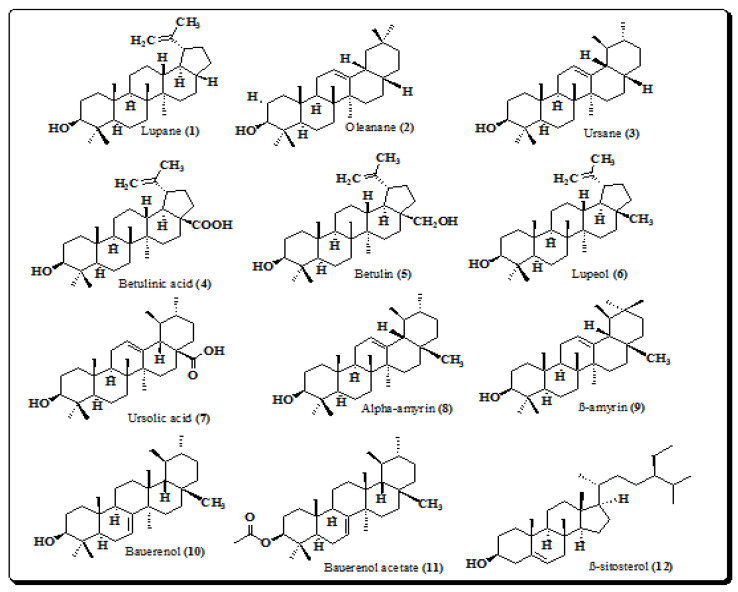
Structures of pentacyclic triterpenoids and phytosterol from *E. laevis* (**1**–**12**).

Pentacyclic triterpenes are abundantly found in medicinal plants and they are synthesized in the cytosol from the cyclization of an epoxidized squalene which is a precursor of the diverse group of polycyclic triterpenes. Terpenes are derived from C_5_ isoprene units, and based on the number of isoprene units, terpenes are classified according to the number of carbon atoms in the polycyclic chain (C_n_) into; hemiterpenes (C_5_) monoterpenes (C_10_), sesquiterpenes (C_15_), diterpenes (C_20_), sesterterpenes (C_25_), triterpenes (C_30_) and tetraterpenes (C_40_). Triterpenoids are either acyclic (only chains without rings or cycles) and pentacyclic (forming five rings or cycles). The pentacyclic triterpenes can be divided into three main classes, depending on the scaffold of their architecture, into; lupane (e.g., betulinic acid, betulin, lupeol) oleanane (e.g., β-amyrin) and ursane (e.g., α-amyrin, ursolic acid), etc. Presently, pentacyclic triterpenes have received much attention because of their versatile biological activities. Pentacyclic triterpenoids are the main active constituents present in its bark and leaves of *E. laevis*. Joshi and Wagh, reported a GC-MS analysis to isolate the triterpenoids such as lupane (**1**), olenane (**2**), ursane (**3**), betulinic acid (**4**), betulin (**5**), lupeol (**6**), ursolic acid (**7**) α-amyrin (**8**), β-amyrin (**9**), bauerenol (**10**), bauerenol acetate (**11**) and β-sitosterol (**12**) from petroleum ether, chloroform and methanolic extracts of its barks and leaves. The structures of most promising triterpenoids are presented in Figure 2. These compounds display various pharmacological actions, and are generally devoid of major toxicity. Therefore, these triterpenes have become the promising leading compounds for the scientific community to design new multi-targeting bioactive agents [9,38].

#### 4.1.1. Betulinic Acid

Betulinic acid (BA, 3β-hydroxy-lup-20(29)-en-28-oic acid), widely distributed throughout the plant kingdom, is a well known pentacyclic lupane-type triterpenoid natural moiety which has gained a lot of attention because of its broad range of pharmacological properties, e.g., antimalarial [42], anti-inflammatory [43], antinociceptive [44], antibacterial [45] and anticancer activities [46]. Besides BA is known to inhibit the growth of canine cancer cell lines and cell arrest in the S-phase of the cell cycle, with an IC_50_ value of 23.5 µM in CL-1 cell line [47,48].

#### 4.1.2. Betulin

Betulin (3β-lup-20(29)-ene-3, 28-diol) is the reduced congener of betulinic acid. It was the first natural moiety isolated from bark of the white birch *Betula alba* in 1788 and its chemical structure was determined in 1952. Betulin exhibits various pharmacological activities, e.g., antimicrobial [49,50], anti-inflammatory and antitumor activities [51]. It has been established that betulin is as potent as betulinic acid towards the CL-1 cell line with IC_50_ value of 27.0 µM and is one of the selective anticancer agents against various human cancer cell lines, e.g., lung cancer, melanoma and lymphoma cells [48,52,53].

#### 4.1.3. Lupeol

Lupeol (lup-20(29)-en-3β-ol) is abundantly found in medicinal plants and has been reported to possess an array of pharmacological activities, including antiangiogenic [54], anti-inflammatory [55], anticancer, antiarthritis, antidiabetic, cardiovascular [56,57,58] and antioxidant activities [59]. Lupeol is one of the potential anticancer biomarkers. It has been investigated that lupeol exhibits anticancer activity against human osteosarcoma cells. It induces apoptosis and cell cycle arrest in G0/G1 phase along with down regulation of PI3-Kinase [59].

#### 4.1.4. Ursolic Acid

Ursolic acid (3β-hydroxy-urs-12-ene-28-oic acid) is a well known pentacyclic terpenoid of plant origin exhibiting a wide range of pharmacological activities, e.g., antiviral [60], antiulcerosos [61], anti-inflammatory and anticancer activities [62].

#### 4.1.5. α-Amyrin

α-Amyrin (3β-hydroxy-urs-12-en) is the precursor of ursolic acid and predominantly found in plant origin, exhibiting an array of pharmacological activities, e.g., anxiolytic, antidepressant [63], anti-inflammatory [64,65], antihyperglycemic and hypolipidemic activities [66]. When administered in a dose of 30 mg/kg to animals, the compound significantly reduced inflammation caused by partial sciatic nerve ligation along with thermal and mechanical hyperalgesia, proving that α-amyrin could be a potential molecule for the relief of pain and inflammation. The compound exerts its extended antinociceptive action through activation of cannabinoid receptors [67].

#### 4.1.6. β-Amyrin

The triterpene β-amyrin (3β-hydroxy-olean-12-en) has shown various pharmacological activities, e.g., antioxidant, anti-inflammatory, analgesic [65], antihyperglycemic and hypolipidemic [66] activities. β-amyrin is also known to exhibit anxiolytic and antidepressant, antimicrobial and antifungal actions [63,68,69,70]. The triterpene β-a both α and β-amyrins are known to minimise the IL-6, TNF-α and IL-1β levels along with the myeloperoxidase activity [71].

#### 4.1.7. β-Sitosterol

The physterol β-sitosterol (3β-stigmast-5-en-3-ol) is one of the important active principles of many plants. It is also used as one of the potential plant biomarkers for the treatment and prevention of cancer [59]. The compound acts by inducing apoptosis and activating caspases such as caspase-3 and caspase-9, with an IC_50_ value of 16 µM in human breast cancer cell lines (MDA-MB-231) [72].

### 4.2. Flavonoids

**Figure 3:**
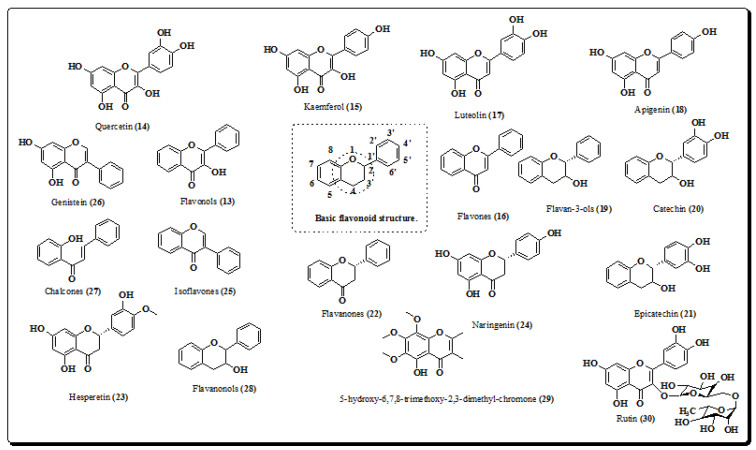
Structures of flavonoids from *E. laevis.* (**13**–**30**).

Flavonoids are a group of natural products, which are ubiquitously present in plants (fruits, vegetables and also in certain beverages) [73]. They are associated with various therapeutic activities and are present in a variety of medicinal, nutraceutical, pharmaceutical, and cosmetic preparations. The basic structures of these compounds are often characterized by a fifteen-carbon skeleton as a common phenyl benzopyrone linkage (C_6_–C_3_–C_6_) in their structures [74]. Flavonoids are a promising class of natural products sub-divided into flavonols (quercetin and kaempferol), flavones (luteolin and apigenin), flavanones (hesperetin and naringenin), flavan-3-ols (catechin and epicatechin) isoflavones (genistein), and flavanones [13,74,75,76]. Structures of flavonoids from the plant (**13**–**30**) are shown in Figure 3.

Flavonoids exert diverse activities, e.g antimycobacterial [77], antioxidant [78], anti-inflammatory [79], anticancer [80,81,82] and antimalarial [83]. Phytochemical screening of methanolic extracts of *E. laevis* indicates the presence of flavonoids [9,10]. The main bioactive molecules isolated from methanolic and its leaf and bark chloroform extracts are flavonoid glycosides. The total flavonoid contents of the plant were determined using the aluminium chloride method [84]. It was also reported that flavonoids (57.23 mg equivalent to rutin (RE)/g) were present in the methanolic extracts of the plant [8]. A quantitative determination of flavonoids isolated from extracts of *E. laevis* using aluminium chloride colorimetric methods showed the presence of rutin and quercetin [10]. The compound 5-hydroxy-6,7,8-trimethoxy-2,3-dimethyl-chromone (**29**) was isolated from the ethyl acetate: formic acid: glacial acetic acid: water fraction [2].

#### 4.2.1. Quercetin

Quercetin (3,3′,4′,5,7-pentahydroxyflavanone) is a citrus polyphenolic flavonoid abundantly present in vegetables and fruits, e.g., black grapes, onion and tea [85,86]. It was the first known tyrosine kinase inhibitor in the phase-I human clinical trials [87,88]. Recent studies have reported for its broad spectrum of activities, including against cancer, cardiovascular diseases, inflammatory and CNS disorders [89,90]. Quercetin exhibits its significant antioxidant activity by sustaining oxidative balance [91]. Antioxidant actions of quercetin are manifested due to its effect on signal transduction, reduced glutathione (GSH) and reactive oxygen species. Quercetin enhances the antioxidant capability of the body by regulating the levels of GSH. It has also been reported that the oral administration of tamoxifen with quercetin encapsulated in nano-particles formulation considerably induces apoptosis and thus attenuating the growth of breast cancer [92,93].

#### 4.2.2. Kaempferol

Kaempferol (3,4′,5,7-tetrahydroxyflavone) belongs to the flavonol class of flavonoid. It is abundantly found in tea, beans, apple, strawberries and spinach [94,95,96]. Recently, numerous investigations established its diverse pharmacological activities, e.g., cardioprotective [97], hepatoprotective [98], anti-inflammatory [99], antioxidant [100], anticancer [101], neuroprotective [102] and antidiabetic properties [103]. Kaempferol was found to be effective against various types of cancers, including skin, colon and hepatic cancer [104,105]. It also has the tendency to scavenge the generation of free radicals viz., hydroxyl, superoxide anions, peroxides and nitric oxide. The anti-inflammatory action of kaempferol has been established in both in vitro and in vivo, and it is known to be via the inhibition of lipopolysaccharide (LPS) and adenosine triphosphate (ATP) and by influencing phosphorylation of AKT and PI3K in cardiac fibroblasts, and thus shielding cells from inflammatory damage [106].

#### 4.2.3. Luteolin

Luteolin (3′,4′,5,7-tetrahydroxyflavone) is a flavone present in a wide variety of fruits, vegetables and in medicinal plants [107,108]. Vegetables including celery, parsley, onion leaves, broccoli, peppers and carrots are rich in luteolin [108]. Luteolin shows an array of biological properties, including antioxidant [109], antimicrobial [108], anticancer [110] and estrogenic regulator properties [111]. Luteolin has the ability to induce apoptosis and produce anticancer effects by causing cell cycle arrest in human oral squamous cancerous cells, human esophageal, colon, lung and liver cancers [112,113,114]. Luteolin also induces apoptosis and inhibits proliferation against human prostate cancer cells in xenografts models [115]. It acts through various mechanisms in cancer including invasion, cell cycle arrest or metastasis by reduction of transcription factors, inhibition of kinases and induction of cell death via apoptosis [116].

#### 4.2.4. Apigenin

Apigenin (4′,5,7-trihydroxyflavone) is predominantly found in everyday diet. Out of all the classes of flavonoids, apigenin is ubiquitous in the plant kingdom. It is rich in tea, oranges, onion, celery, parsley, beer and wines [117]. Apigenin attracts researchers and has been recommended in nutraceuticals because of its numerous benefits and low toxicity [118]. Apigenin exhibits a broad spectrum of activities and is used in the cure of amnesia, depression, stroke, diabetes and cancer [119,120]. Numerous in vitro and in vivo studies support the therapeutic potential of apigenin as antioxidant, anti-inflammatory and anticancer [120]. It induces apoptosis by activating caspase-3, release of cytochrome-C in cytoplasm, reduction of mitochondrial membrane potential loss [121,122]. Antidepressant and neuroprotective effects of apigenin were observed, as well as its intervention on lipopolysaccharide (LPS) induced depressive-like behavior in animal models of mice [123]. The antidiabetic action of this compound has been established due to its ability to inhibit the α-glucosidase activity, resulting in increased release of insulin [124]. Apigenin also exhibits its anti-HIV activity in T-cell line (H9) contaminated with HIV-I and HIV-1 (IIIB) [125,126,127].

#### 4.2.5. Naringenin

Naringenin [5,7-dihydroxy-2-(4-hydroxyphenyl)chroman-4-one] belongs to the flavanone series of flavonoids and is predominantly found in citrus fruits like oranges, lemons, grapes and tomatoes. It is a common polyphenolic dietary component and is derived from the hydrolysis of narirutin or naringenin-7-rutinoside [128,129]. The scientific community pays considerable attention to this flavonoid because of its therapeutic potential, including its antioxidant [130], antidiabetic [131], and anti-inflammatory properties [132] and potential against malignancies and neurodegenerative diseases [133,134]. Naringenin exerts its antioxidant effects by scavenging free radical generation and enhancing several antioxidant enzyme levels such as glutathione peroxidase, catalase and superoxide dismutase [135]. It also has potential anticancer properties against breast cancer MDA-MB-231 cell lines by inhibiting HER2-TK activity, in prostate cancer by mitochondrial membrane potential loss, and in liver cancer via activation caspase-3 and induction of apoptosis [136,137,138,139]. Naringenin also exhibits antidiabetic activity in vitro at a dose of 5 µg and in vivo at the dose of 50 mg/kg by decreasing the glucose level [140,141].

#### 4.2.6. Rutin

Rutin (3′,4′,5,7-tetrahydroxyflavone-3-rhamnoglucoside) is abundantly available as a flavonol of plant origin. The compound is abundantly present in fruit skin, buckwheat and potato skin of this plant [142]. It exhibits various pharmacological activities including neuroprotective [143], cardioprotective [144], antidiabetic [145], anticarcinogenic [146], anti-inflammatory [145], and antioxidant [146,147,148,149,150,151]. It scavenges free radicals and inhibits the lipid peroxidation [152,153]. It is also reported to act as a hepatoprotective agent [154].

### 4.3. Phenolic Acids and Tannins

Plant phenolic acids are a fundamental human dietary component and are well renowned for their pharmacological actions such as antioxidant [155], anticancer [156], antiallergic [157], antimicrobial [157] and anti-inflammatory properties [157,158]. The antioxidant potential of a particular phenolic acid depends on the number of hydroxyl groups present as well as their position on the molecule. Tannins belong to the class of polyphenols. Tannins are water soluble compounds, are present in many plants and have the ability to precipitate proteins [159,160,161]. Polyphenols are considered to be significant antioxidants and also act as therapeutic candidates in the mitigation of many diseases. Gallic acid and tannic acid are the main phenolic acids present in leaves and stem bark of this plant [8,84,162].

#### 4.3.1. Gallic Acid

**Figure 4:**
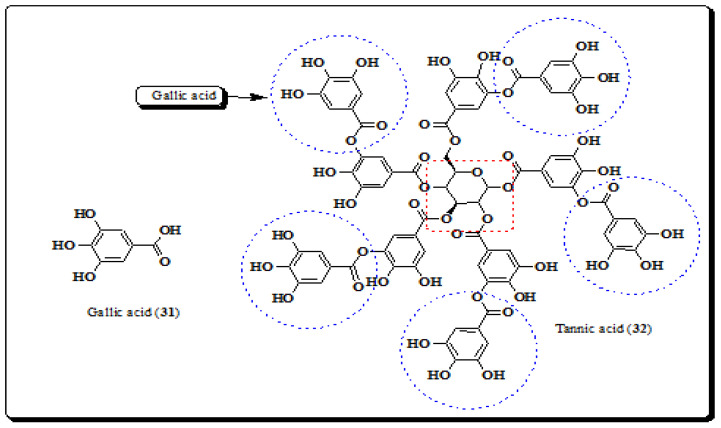
Structures of polyphenolic compounds from *E. laevis.* (**31**, **32**).

Gallic acid (3,4,5-trihydroxybenzoic acid) is a naturally-occurring plant phenol obtained by the hydrolysis of tannins. Gallic acid (**31**) is known for its diverse biological activities such as, hepatoprotective [163], anticancer [164], antimicrobial [165] and gastrointestinal disorders [166,167]. Oxidative stress results in an accumulation and overproduction of free radicals, and is the foremost origin of several degenerative diseases such as cardiovascular system (CVS) diseases [167], atherosclerosis [166], cancer [164] and inflammatory diseases [168]. Gallic acid (Figure 4) is a low molecular weight compound readily available in fruits, vegetables and medicinal plants. It has the ability to induce apoptosis and also acts as a strong antioxidant. It has been found in the methanolic extract of leaves of *E. laevis* [8,157,158].

#### 4.3.2. Tannic Acid

Tannic acid (1,2,3,4,6-penta-*O*-{3,4-dihydroxy-5-[(3,4,5-trihydroxybenzoyl)oxy] benzoyl}]-D-glucopyranose) (**32**) is a polyhydroxy phenol, whose structure contains a large number of phenol units, Figure 4 [169,170]. The phytochemical investigation of the stem bark and leaves of *E. laevis* reported the existence of tannic acid, along with other phytoconstituents in noticeable quantities. Rangnathrao and Shanmugasundar identified tannic acid by preliminary phytochemical screening of methanolic extracts of the stem bark of *E. laevis* and showed that this contains a high concentration of tannins, 64.12 mg of tannic acid equivalent (TAE)/g [8,10].

### 4.4. Amino Acids

**Figure 5:**
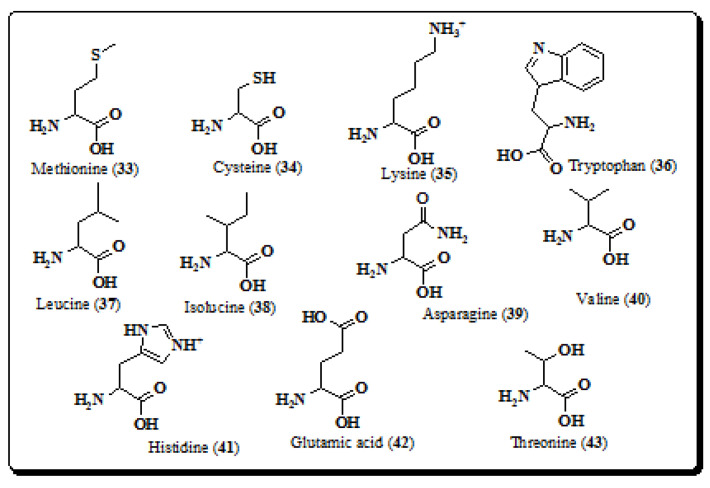
Structures of amino acids from *E. laevis.* (**33**–**43**).

Amino acids are the building blocks or basic units of proteins, which compose the foremost part of our body weight. They play an important role in our body since they are essential for vital processes such as synthesis of neurotransmitters and hormones [171]. Velappan and Thangaraj established the amino acid profiles of edible parts of *E. laevis* and compared it with reference levels of amino acids [8,172], showing that methionine (**33**), cysteine (**34**) and lysine (**35**) are most abundant amino acids of barks and leaves of *E. laevis*, whereas fruits are rich in tryptophan (**36**), leucine (**37**) and isoleucine (**38**). Additionally, asparagine (**39**), valine (**40**), histidine (**41**), glutamic acid (**42**), and threonine (**43**) were presented in traces [8,172]. The chemical structures of the identified amino acids are represented in Figure 5.

### 4.5. Carbohydrates

**Figure 6:**
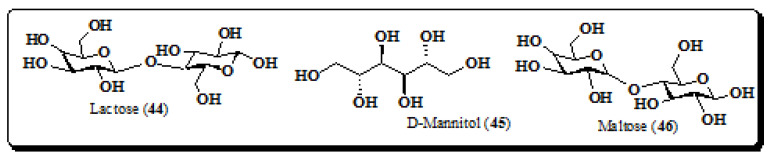
Structures of carbohydrates from *E. laevis* (**44**–**46**).

The phytochemical investigation of the stem bark and leaves of *E. laevis* revealed the presence of primary metabolites such as carbohydrates in an appreciable quantity. Three carbohydrates (Figure 6) namely lactose (**44**), D-mannitol (**45**) and maltose (**46**) (Figure 6) were identified from the leaves [6,173,174,175].

### 4.6. Vitamins

**Figure 7:**
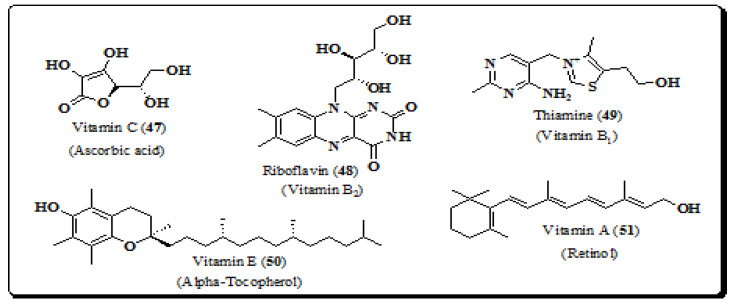
Structures of vitamins from *E. laevis* (**47**–**51**).

Literature on the quantitative assessment of trace elements in the leaves of *E. laevis* establish its nutritional value due to the presence of minerals and vitamins such as vitamins C, E, A, riboflavin and thiamine (**47**–**51**), Figure 7 [22,34]. Vitamin C plays a significant role in slowing the development and prevention of several diseases by exhibiting antioxidant action by scavenging free radicals and also acting as an enzyme cofactor in cells [176,177,178,179,180,181].

### 4.7. Minerals

Minerals are one of the essential and vital components of food and fodder. All the minerals play an important role in the structural and metabolic activities of the body, e.g., brain development, gastrointestinal tract (GIT) functions, bone development, bones and teeth strength. The fruits and inner bark of *E. laevis* are reported to be edible [32,182]. Experimental analysis of the bark, fruits and leaves of *E. laevis* confirmed the presence of a significant amount of minerals (Table 2), e.g., sodium (2.12 g/kg), phosphorous (7.36 g/kg) and calcium (38.31 g/kg) found to be abundant in the leaves, while a few minerals like silica (5.21 g/kg), copper (0.05 g/kg) and zinc (3.16 g/kg) were obtained from stem bark. The fruits of *E. laevis* were found to have notably high quantities of potassium (28.12 g/kg), manganese (0.04 g/ kg), magnesium (13.45 g/kg), and iron (1.28 g/kg) [8,13,183].

### 4.8. Miscellaneous

**Figure 8:**
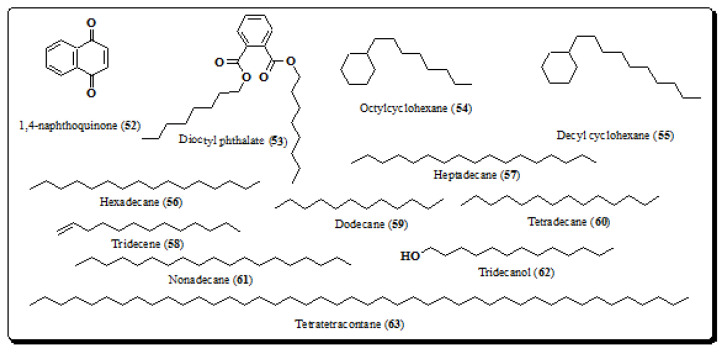
Structures of other compounds from *E. laevis* (**52**–**63**).

Other minor compound classes like 1,4-naphthoquinone, dioctyl phthalate, aliphatic hydrocarbons, fatty acids, esters and benzofurans have also been isolated from different extracts of *E. laevis*. Lewisone (1,4-naphthoquinone, **52**) is one of the important benzoquinones involved in the synthesis of vitamin K (Figure 8). Compound **52** is a new naphthoquinone from the aerial parts of *E. laevis* and showed significant antibacterial, antifungal, antiviral and anti-inflammatory properties [41]. Several other compounds, e.g., dioctyl phthalate (**53**), octylcyclohexane (**54**), decyl cyclohexane (**55**), hexadecane (**56**), heptadecane (**57**), tridecene (**58**), dodecane (**59**), tetradecane (**60**), nonadecane (**61**), tridecanol (**62**) and tetratetracontane (**63**), shown in Figure 8, have also been isolated from the bark of *E. laevis* [8,41].

**Figure 9:**
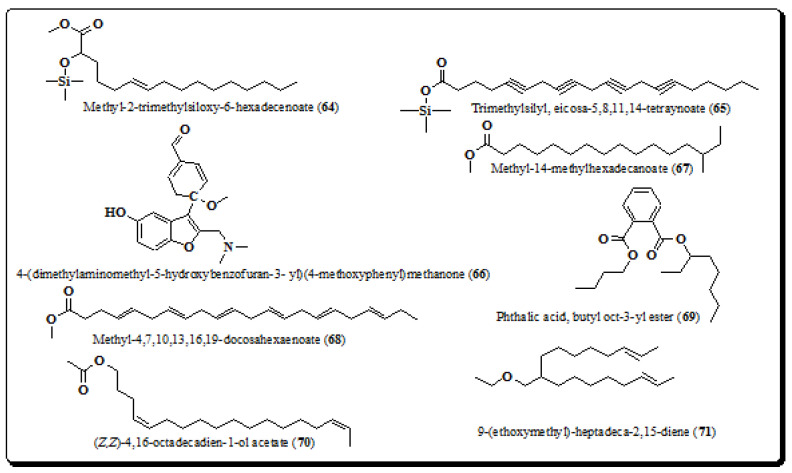
Structures of compounds from *E. laevis* (**64**–**71**).

Some of the bioactive constituents have also been isolated from methanolic extract from the bark of *E. laevis*. The structures of isolated compounds are methyl ester of 2-trimethylsiloxy-6-hexadecenoate (**64**), trimethylsilyl-eicosa-5,8,11,14-tetranoate (**65**) and 4-(dimethylaminomethyl-5-hydroxybenzofuran-3-yl)(4-methoxyphenyl) methanone (**66**), Methyl-14-methylhexadecanoate (**67**), Methyl-4,7,10,13,16,19-docosahexaenoate (**68**), Phthalic acid, butyl oct-3-yl ester (**69**), *Z*,*Z*-4,16-octadecadien-1-ol acetate (**70**) and 9-(ethoxymethyl)-heptadeca-2,15-diene (**71**), Figure 9 [9].

**Figure 10:**
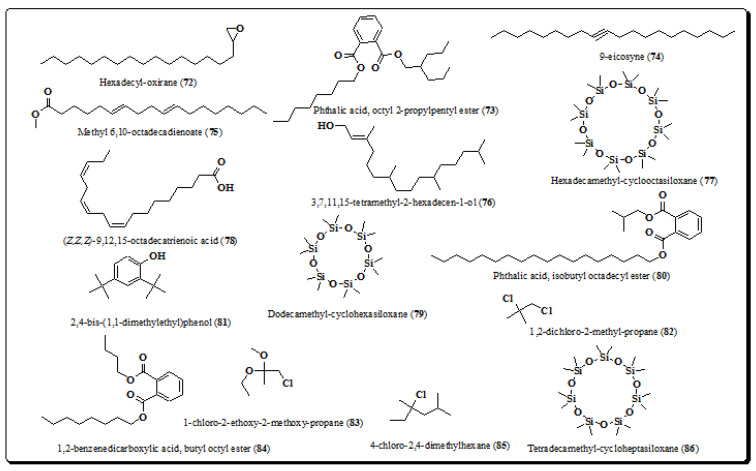
Structures of compounds from *E. laevis* (**72**–**86**).

Joshi and Wagh identified fifteen compounds, including hexadecyl-oxirane (**72**), phthalic acid, octyl 2-propylpentyl ester (**73**), 9-eicosyne (**74**), methyl-6,10-octadecadienoate (**75**), 3,7,11,15-tetramethyl-2-hexadecen-1-ol (**76**), hexadecamethyl-cyclooctasiloxane (**77**), (*Z*,*Z*,*Z*)-9,12,15-octadecatrienoic acid (**78**), dodecamethyl-cyclohexasiloxane (**79**), isobutyl octadecyl ester (**80**), 2,4-bis-(1,1-dimethylethyl)phenol (**81**), 1,2-dichloro-2-methyl-propane (**82**), 1-chloro-2-ethoxy-2-methoxy-propane (**83**), octyl ester of 1,2-benzenedicarboxylic acid (**84**), 4-chloro-2,4-dimethylhexane (**85**) and tetradecamethyl-cycloheptasiloxane (**86**) (Figure 10) from chloroform extract of bark of the plant [9,184].

**Figure 11:**
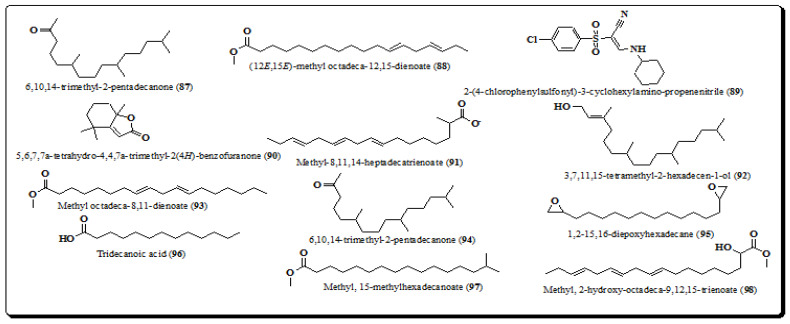
Structure of other compounds from *E. laevis* (**87**–**98**).

Furthermore, using GC-MS profiling analysis established the structure of long chain aliphatic esters, alcohols, ketones, carboxylic acids and various other compounds (Figure 11), from petroleum ether extract of bark of the plant. These include 6,10,14-trimethyl-2-pentadecanone (**87**), (12*E*,15*E*)-methyl octadeca-12,15-dienoate (**88**), 2-(4-chlorophenylsulfonyl)-3-cyclohexylamino-propenenitrile (**89**), 5,6,7,7a-tetrahydro-4,4,7a-trimethyl-2(4H)-benzofuranone (**90**), methyl-8,11,14-heptadecatrienoate (**91**), 3,7,11,15-tetramethyl-2-hexadecen-1-ol (**92**), methyl octadeca-8,11-dienoate (**93**), 6,10,14-trimethyl-2-pentadecanone (**94**), 1,2-15,16-diepoxyhexadecane (**95**), tridecanoic acid (**96**), 15-methylhexadecanoate (**97**) and 2-hydroxy-octadeca-9,12,15-trienoate (**98**) [185,186].

A variety of carbohydrates, proteins, minerals, amino acids and vitamins are present in *E. laevis*, making the plant good for food and fodder. Ripe fruits of plants are eaten by local children [187,188,189,190]. Numerous research findings have established the presence of other classes of phytoconstituents in all parts of plant, which are detected by qualitative phytochemical screening. These include alkaloids, phenolics, saponins, flavonoids, triterpenoids, glycosides, tannins and amino acids. However, more detailed studies should be carried out in order to further isolate and explore the new bioactive metabolites from *E. laevis*, which could become a potential therapeutic candidate for the treatment of diverse disorders.

## 5. Pharmacological Reports

A wide variety of phytoconstituents are present in *E. laevis*, many of which exhibited an array of pharmacological activities. Several ethnobotanical survey studies reported the characteristic uses of the plant in the treatment of ailments, including jaundice and liver diseases, chronic and acute inflammations, ulcers and gums problems, wound healing and pains [15,16,18,19,20,21]. This part of the review will emphasize on its pharmacological studies reported for *E. laevis* extracts, fractions and of its phytoconstituents, along with their ethnopharmacological relevance.

### 5.1. Anti-inflammatory, Antiarthritic and Analgesic Activities

An ethnobotanical survey revealed that the tribes of rural and forest remote areas are still depending to a great extent on indigenous system of medicine [5,75,127]. The community of Jalpaiguri district, West Bengal local people apply the paste from the bark of *E. laevis* to treat painful limbs [19]. The bark juice of the plant can also be used traditionally in obstetric practice for the relief of delivery pain [18]. The plant has been recommended as an ethnic remedy for pain and inflammation. In the community of Amravati District, Maharashtra, the people also apply the root extract for the cure of inflammation [15]. Recently, in vivo studies established the anti-inflammatory potential of methanol, chloroform and aqueous extracts of *E. laevis* [31,191], including its potential for the treatment of arthritis, a condition characterized by chronic inflammation [192]. Besides, the methanolic extract of *E. laevis* leaves has been investigated for its antiarthritic activity in induced arthritis models in rats [193,194,195]. Phytoconstituents like hexadecanoic acid (palmitic acid), oleanolic acid, and other fixed oils were suggested to be responsible for its antiarthritic actions. Although systematic scientific studies are still lacking, forthcoming work will probably produce interesting consequences and may provide a prospective remedial candidate from *E. laevis* for the treatment of inflammatory disorders [196,197].

### 5.2. Antioxidant Activity

Several studies suggest the antioxidant potential of plant *E. laevis*. Antioxidants are the substances which have capacity to inhibit or delay the oxidation process under the influence of either reactive oxygen species or environmental oxygen [198]. Antioxidants are compounds which protect living organisms from damage caused by concomitant lipid peroxidation, protein damage; uncontrolled ROS production and breaking of the deoxyribonucleic acid (DNA) strand [199]. In Ayurveda, there are many plants that possess antioxidant potential and can be used against diseases in which free radicals and ROS play an important role [200]. Ethnomedical literature reports reveal that the plant. *E. laevis* contains compounds like ascorbic acid, phenolic acids, flavonoids, carotenoids and polyphenolic acids, which have the tendency to scavenge free radicals such as hydroperoxide, lipid peroxyl or peroxide and thus hamper oxidative stress that causes degenerative diseases [201].

Various in vitro studies account for antioxidant potential of the plant *E. laevis*. The antioxidant effect of bark extracts of *E. laevis* was investigated using 2,2-diphenyl-1-picrylhydrazyl (DPPH) radical scavenging, hydrogen peroxide, nitric oxide radical scavenging and reducing capacity assay. The methanolic and hydroalcoholic extracts scavenged DPPH radical (87.13% and 72.35%), nitric acid (85.08% and 74.74%) and hydrogen peroxide (87.55%, 76.39%) at 200 µg/mL. The flavonoids and phenolic contents were determined using aluminium chloride colorimetric assay and Folin–Ciocalteu’s method respectively. Investigators quantified total flavonoids and phenolic contents from methanolic extract were found to be 78.75 mg/g in terms of the quercetin equivalent and 89.55 mg/g in terms of the gallic acid equivalent respectively [9,202]. A study of the antioxidant activity of leaf extract of *E. laevis* showed that it exhibited significant antioxidant activity with a minimal inhibitory concentration (MIC) value of 0.02 mg/mL [203]. Another study investigated the antioxidant capability of the leaves and stems of *E. laevis*, in which the ethanolic extracts of the leaves and stem were subjected to DPPH scavenging activity [204]. This revealed that the antioxidant ability of ethanolic extract of leaves and stem, measured by DPPH radical scavenging power, showed IC_50_ values 2.44 and 29.88 μg/mL, respectively. The total flavonoid and phenolic contents of ethanolic extracts of leaves and stem *E. laevis* were calculated to be 18.19 and 1.29 mg/g in terms of quercetin equivalent and 19.02 mg/g and 7.84 mg/g in terms of catechol equivalent, respectively [204].

In another study, the methanolic extract of *E. laevis* fruits was reported for antioxidant effects [37]. The plant displayed its antioxidant effects due to the presence of phytomolecules such as flavonoids, tannins, ascorbic acid and phenolic acids. In vitro antioxidant activity of methanolic extract of fruits have been established using DPPH, hydrogen peroxide, hydroxyl radical and nitric oxide scavenging assays, along with ferrous chelating assays. The methanolic extracts showed DPPH, hydrogen peroxide, hydroxyl and nitric oxide radical scavenging activities, with IC_50_ values of 122.56 µg/mL, 112.02 µg/mL, 14.86 µg/mL and 21.09 μg/mL, respectively [37]. The utilization of plants containing antioxidants has been recommended in the management of several diseases in view of the fact that oxidative stress is an outcome of free radical production which occurs through cellular respiration in body has been engaged in the pathogenesis of various human diseases, including ischemia, parkinson’s disease, schizophrenia, cancer, diabetes, huntington’s disease, inflammation, epilepsy, arthritis, anxiety, gastritis, atherosclerosis, senile dementia, depression and asthma [205,206,207,208]. The antioxidant potential of various other parts of *E. laevis* needs to be explored further in ameliorating the oxidative stress associated disorders.

### 5.3. Antimicrobial Activity

*E. laevis* has been employed as an ethnic medicine for the treatment of several infectious diseases, including those of viral, fungal, protozoal and bacterial origin. Several investigations have been performed in the recent past years to authenticate the antimicrobial potential of *E. laevis*. For example, the plant has been tested against different Gram-positive and Gram-negative bacterial strains. The methanol, chloroform, and aqueous extracts showed inhibition of *Staphylococcus aureus*, *Pseudomonas aeruginosa*, *Bacillus subtilis* and *Escherichia coli* strains using the agar well diffusion assay [209,210,211]. The results reveal that all the extracts have significant antibacterial action against Gram-negative and Gram-positive bacteria, establishing that the plant has a broad spectrum of activities. Antibacterial activities of the methanol, chloroform, and aqueous extracts at different concentrations (250, 500 and 1000 µg/mL) inhibited the growth of all bacterial strains. The methanolic extract showed noteworthy antibacterial action against *B. subtilis* and *P. aeruginosa* [31,75].

Another study confirmed the antimicrobial action of methanolic extract of *E. laevis* against salivary microflora using agar well diffusion method at 400 and 800 µg/mL [212]. The methanolic extract inhibits the tested microorganisms with 4.8 and 6.7 mm of zone of inhibition respectively after 24 h [213]. Recently, the antimicrobial activity of isopropanol and acetone extracts of *E. laevis* leaves have been evaluated against *Pseudomonas aeruginosa, Escherichia coli* and *Staphylococcus aureus*. The results established that the extract was considerably effective towards *S. aureus* and *E. coli*. The antimicrobial potential was found in terms of minimum inhibitory concentration (MIC) using agar broth dilution assay. The MIC of isopropanol extract was 1 mL and 2.5 mL, and for acetone extract was 1.25 mL and 2.5 mL against *E. coli* and *S. aureus*, respectively. All these findings confirm the traditionally claimed antimicrobial potential of *E. laevis*. There is always a tremendous demand for antimicrobial agents due to the speedy development of microbial resistance. The bioactive constituents of this plant could be excellent lead compounds in the search of new potential antimicrobial agents [13,214,215].

### 5.4. Wound Healing Activity

A tribe of Wardha district of Maharashtra, India used *E. laevis* for the management of wound healing and found interesting results [216]. Similarly, folklore practitioners of Garasia community of district Sirohi, Rajasthan also recommended the paste prepared from leaves of plant for early healing of cuts [18]. Thakre et al. reported the wound healing activity of paste made from leaves of *E. laevis* [217]. Investigators used paste in thirty-four patients and scrutinized the patients based on several parameters such as different age groups, gender, fresh, chronic, infected and non-infected. According to Bates-Jansen wound assessment, a specified quantity of paste has been applied after an interval of seven days [217]. The results revealed that wounds were healed completely from minimum of seven days to maximum sixty-six days in all the patients except one [217].

Recently, a case report had been published on the local application of *E. laevis* (Khanduchakka Ghrit) in the treatment of anal fissure (Parikartika) [218,219]. It has been observed that after all the mandatory measures, the efficacy of the plant has been evaluated on the basis of clinical parameters such as bleeding, itching, pain and healing. Patients were found to be healed with no signs of bleeding, pain and itching after twenty days of local application on rectum fissure. The procedure of application was followed twice a day for the duration of one month. The assessments were carried out at 0, 7, 14 and 21 days. The follow up was also conducted after 30 and 45 days. Even during follow up assessment no signs of recurrence were reported [220]. A broad antimicrobial spectrum of barks and leaves can be a probable rationale for its wound healing property. Till now, no investigational work is presented on the wound healing activity as well as in the management of anal fissures. So, there could be a wide scope for future research to figure out the possible mechanism and possible phytoactive metabolites for wound healing effects.

### 5.5. Dental Caries

Dental caries is a foremost health trouble of oral cavity. Dental caries is initiated by the interaction of microbes on the tooth enamel [221]. It is anticipated that about 2.3 billion inhabitants suffer from dental caries globally. According to the World Health Organization (WHO) the incidences of dental caries are constantly increasing. It affects all races, genders and age groups. The prevalence of caries is about 49% before the age of 12 years, while it progressively increases from 15 years (60%) and peaks at the age group of 60–74 (84%) [222,223]. Salivary microflora is mainly accountable for dental caries. This salivary microflora contains cariogenic microorganisms which are involved in the process of caries formation and also perturb the normal microflora of oral cavity [224]. An ethnomedicinal survey carried out by Patil and Patil, found that the tribe of Dhule district of Maharashtra used the stem of *E. laevis* as a brush for the cure of ulcers of mouth and gum problems [16]. Similarly, another study reported that people living in remote areas of Rajasthan chewed the leaves of *E. laevis* to treat blisters of mouth [24]. The remote areas of Pakistan also use this plant for the cure of dental caries [225]. It has also been documented that several parts of the plant were used for oral fitness [226]. Young branches of *E. laevis* were also employed for the relief of toothache [35]. Recently, Deshpande et al. investigated that the methanolic and ethanolic extracts of *E. laevis* inhibited the zone of inhibition towards salivary microflora at different concentrations (50, 100, 200, 400 and 800 µg/mL). It has been found that the methanolic extract at 100 µg/mL showed feeble antimicrobial action, whereas the zone of inhibition was increased up to 8.4 mm at 800 µg/mL [227]. All these documented reports validate the traditional uses of *E. laevis* towards microbial diseases of oral tissues. A wide antimicrobial spectrum of *E. laevis* can be very helpful in the prevention of dental problems. Therefore, all these studies confirmed that *E. laevis* has the potential for the therapeutic management of oral/dental problems.

### 5.6. Miscellaneous Activities

*E. laevis* had also been studied for its coagulant and edible properties. Moreover, several other parts of the plant were recommended for the treatment of jaundice, skin diseases and in bone fractures. In another ethnobotanical survey the folklore practitioner of Uttarakhand recommended the paste prepared from the seeds of *E. laevis* mixed with cardamom powder and to be administered with milk three times a day for the management of jaundice and in other liver problems [20]. Recently, the coagulant potential of extracts of *E. laevis* leaves has been evaluated, revealing that the coagulant potential of isopropanol extract is more significant than the acetone extract [228].

*E. laevis* has also been used by tribal people and recommended by folklore hakeem for the management and healing of fractures. Tichkule et al. reported the use of paste prepared from the leaves of *E. laevis* for the management of fracture. The kalka (paste) was applied in the form of lepan (applied locally) at the affected sites [14,229]. The dressing is applied daily then covered by rubber pads and support has been provided using slabs of plaster of paris. A bandage was applied over the whole part of the foot and leg. After the application of lepan for 10 days, it was found that the swelling and pain had been abridged gradually and patients became able to move without any kind of support. The assessment of swelling was done using the American Orthopedic Foot and Ankle Scale (AOFAS) [230]. The X-ray reports of patients revealed that the application of lepan of the plant over the fractured portion for two weeks lead to callus formation and unite malunion of metatarsal. Sharma, et al. established that the Tharu community of Uttrakhand used the ground paste of leaves of *E. laevis* has been applied topically for the treatment of diverse skin disorders [18,21,33]. In another study, the antidiabetic perspective of *E. laevis* has been established through electrochemical measurement using multi-walled nano carbon tubes [76,231].

## 6. Toxicology

The therapeutic perspective of the plant or isolated molecule is considered commendable when it is devoid of any sign of adverse effects or toxicity. It is well acknowledged that toxicity studies are of most imperative for natural products and also for their isolated compounds. Even though numerous pharmacological activities of *E. laevis* have been established to support its pervasive traditional and customary use of the plant as antioxidant, analgesic, anti-inflammatory, antimicrobial, antiarthritic, and also used in several liver, skin, inflammatory, dysentery, infectious, and dental problems. None of the research groups approved any toxicity of the plant. Moreover, the plant is widely accepted as food, as its ripened fruits are eaten by tribal people. Only one report on acute oral toxicity study has been performed as per the organization for economic cooperation and development (OECD) guidelines 423. All the methanolic extracts of leaves, stems and fruits were considered safe at the dose of 2000 mg/kg [8]. Assessment of body weight and behavioral changes were also observed. There was no sign of toxicity (mortality), as all these three extracts showed no neurotoxic effects and were found to be safe [232,233].

The toxicity reports on *E. laevis* are very limited and inadequate to support the efficacy and safety of its extracts/fractions in different ailments, which may lead to delay its use as a therapeutic candidate. Likewise, with available literature reports are deficient in the scientific knowledge and toxicity of active constituents of plants. The interaction of extracts with pharmaceutical additives/drugs needs to be studied at a methodological level. It is also important to mention here that detailed pharmacodynamic and pharmacokinetic studies of the plant (*E. laevis*) extracts have not been investigated yet in clinical trials to transform it into its remedial use.

## 7. Discussion

*E. laevis* is one of the famous plants used by various tribes of tropical Asia, Africa and Australia in ethnomedical treatment of jaundice, diarrhea, cough, syphilis, dysentery, asthma, fissure, dental, skin, infectious and liver diseases [8]. Pharmacological investigations carried out on various plant parts, crude extracts/fractions and isolated molecules of *E. laevis* grant a realistic support for its conventional uses. Recent studies have been paying attention to the investigation of antioxidant, analgesic, anti-inflammatory, antiarthritic, antimicrobial, wound healing activities. Most of the performed pharmacological activities were intended to authenticate its customary uses. Several traditional uses have been explored by various investigators. Its therapeutic use for dental problems, treatment of fissure, skin diseases, diarrhea, dysentery, cuts and wound healing were supported by its antimicrobial potential. *E. laevis* has been used historically in inflammation, arthritis and liver problems. These traditional claims have been supported by its in vitro antioxidant activities. All these pharmacological activities have been evaluated by using various assays. Its pentacyclic triterpenoids, flavonoids and phenolic compounds may be responsible for the above-mentioned activities. Further investigations are still required to be carried out to identify the possible mechanism and to pinpoint the bioactive phytoconstituents of *E. laevis* responsible for mitigation of various diseases.

Quantitative analyses have been carried out by the researchers led to the investigation of a few classes of bioactive phytochemicals. However, pentacyclic triterpenoids and phenolic compounds got attention by several researchers [7,9]. No research group had isolated active constituents such as alkaloids and saponins. Despite numerous research groups signify the presence of these classes of phytoconstituents in all parts of plant by qualitative phytochemical screening [6,7,8,9,85]. Further detailed phytochemical studies need to be carried out to isolate and to explore the new bioactive metabolites from *E. laevis* so that these may emerge as potential therapeutic candidates for the treatment of diverse disorders. Additionally, till today clinical and preclinical studies have not been performed in detail to support its efficacy and safety in rodents as well as in humans. This review article is very helpful for the scientific community as it provides insights for reviewers, readers, researchers and academicians who are working in the area of phytochemistry to understand *E. laevis* perspectives for in depth investigations.

Nevertheless, the enormous customary uses and proven pharmacological activities of *E. laevis* reveal that a wide scope still subsists for its phytochemical investigation. Several studies described sound pharmacological activities of *E. laevis*, which could be investigated further for maximum utilization of this plant as a remedy for various ailments. The plant contains flavonoids and amino acids like tryptophan, asparagine, glutamic acid, histidine etc. The use of plant-based amino acids have been recommended to alleviate in tumor, liver and neuropsychiatric complications. Hence, this plant can also be explored for cancer, liver and central nervous system (CNS) disorders [20,34,80,233].

The present review compiles published information on the isolated phytoconstituents, reported activities and toxicity of *E. laevis*. Authors have tried to summarize relevant data on phytochemistry including the structures of isolated molecules from different extracts, pharmacological evaluation, folklore uses and common names of the plant in different languages (see Supplementary Materials, Table S1). This work highlighted up to date records and gaps in research, which exists for this plant to categorize it as a prospective ethnopharmacological and potential therapeutic plant.

## 8. Conclusions

This review presents the ethnobotanical description, ethnopharmacological uses, bioactive phytometabolites and pharmacological properties of *E. laevis*. This plant exhibited fascinating therapeutic perspectives. However, all the reported pharmacological studies were limited and scientifically incapable to validate it as a potential candidate for the cure of ethnomedical disorders. Up till now, no clinical or detailed preclinical investigations had been carried out to endorse its safety. Sporadic phytochemical work was carried out to isolate its active phytoconstituents, following the bioactivity-guided fractionation approach. The available literature also reveals that no work has been performed towards standardization of plants. Though the plant possesses significant scientific and traditional data to justify its medicinal value, modern approaches like molecular docking, quantitative structure-activity relationship studies and development of leads from *E. laevis* are completely missing. The article presents the gigantic gaps in research investigations done on *E. laevis*. In addition, detailed preclinical and clinical trials ought to be explored in future to evaluate *E. laevis* clinical effectiveness and safety in humans. The results of the upcoming investigations on the above-mentioned areas will certainly offer persuasive support for the future clinical significance of *E. laevis* in modern medicine.

## Figures and Tables

**Table 1:** Local and contemporary uses of *E. laevis*.

S. No.	Ailment/Use	Part of Plant	Locality/System of Medicine	Preparation Used	References
1	Abdominal pain	All parts of plants	Tribal of Wardha district of Maharashtra	Decoction and juice	[5,11]
2	Acute and chronic inflammations	Root	Korku tribe	Root extract	[15]
3	Analgesic	Bark	Jalpaiguri/West Bengal	The paste of bark has been used for the relief of pain especially for lower limbs.	[19]
4	Anthelmintic	Fruits and seeds	Asia and Australian tropics/Pune (Maharashtra)	Decoction	[2,23]
5	Antidote to vegetable poison	Stem bark, leaves and fruits	Tropical Asia and Australian tropics/Krishnagiri District, Tamil Nadu, India	Unknown	[5]
6	Antivenom (Vishaghna)	Leaves	Ayurveda	Unknown	[13]
7	Aphrodisiac	Powder of flowers	Tropical Asia and Australian tropics/Krishnagiri District, Tamil Nadu, India	Powder of flowers with milk has been employed as an aphrodisiac.	[5]
8	Asthma	Leaves and bark	Garasia tribes/Rajasthan	Decoction, juice of leaves and bark juice.	[18]
9	Astringent	Fruits	Asia and Australian tropics/Pune (Maharashtra)	Juice of fruits	[2,23]
10	Blisters of mouth	Leaves	Remote areas of Rajasthan	Powdered leaves were chewed with equal quantities of sugar.	[24,25]
11	Cachexia	Stem bark, leaves and fruits	Tropical Asia and Australian tropics/Krishnagiri District, Tamil Nadu, India	Unknown	[5]
12	Cough and cold	Leaves and bark	Garasia tribes/Rajasthan	Decoction of leaves and bark juice.	[18]
13	Cuts and wounds	Leaves	Garasia tribes/Rajasthan	The leaves were grounded and its paste is applied topically on wounds.	[18,21]
14	Delivery pain	Bark	Garasia tribes/Rajasthan	Bark juice	[18]
15	Demulcent	Fruits	Asia and Australian tropics/Pune (Maharashtra)	Unknown	[2,23]
16	Dental caries	Stems	Ayurveda	Bark is used as toothbrush	[26]
17	Diabetes (Prameha)	Leaves	Ayurveda	Decoction	[13]
18	Diarrhea	Bark and roots	Ayurveda	Decoction of bark and roots	[5]
19	Diphtheria	Stems and bark	Ayurveda	Decoction of stem and bark for the treatment of diphtheria.	[5]
20	Diuretic	Fruit	Asia and Australian tropics/Pune (Maharashtra)	Decoction	[2,23]
21	Dysentery	Stem bark	South West Bengal/district—Dindori, Madhyapradesh	Stem bark powder has been administered orally thrice in a day for the treatment of dysentery.	[27,28]
22	Dysuria	Leaves	Kota district of Rajasthan	Leaf powder was mixed with sugar and divided into 10 equal doses. Each dose has been administered daily orally along with goat milk or curd for cure of dysuria.	[25]
23	Eczema	Leaves	Tharu community/Uttarakhand	Paste of leaves was applied topically for the cure of eczema.	[21]
24	Expectorant	Fruit	Asia and Australian tropics/Pune (Maharashtra)	Decoction	[2,23]
25	Fever	Leaves	Garasia tribes/ Rajasthan	Decoction of leaves	[18]
26	Fissure	Leaves	Wardha Tribe/Maharashtra	*Ghrit* was prepared by using fresh leaves and applied locally on anal fissure twice a day for 21 days. Additionally, *Triphala Churna* (10 g) has also been recommended before bed time for one month with lukewarm water.	[17]
27	Food and fodder	Bark, leaves, stems and fruits	Tropical Asia and Australian tropics/Krishnagiri District, Tamil Nadu, India.	Inner bark of *E. laevis* has been used as food (dietary supplement for humans and cattle). Fruits are also edible by tribal children.	[5,23]
28	Fracture	Leaves	Ayurveda/ Garasia tribes/Rajasthan	*Kalka* (paste) was prepared and applied all over the foot. Cotton pad and cotton roll were applied firmly. The thickness of *Kalka* was 0.5–1 cm. This *Kalka Lepana* was kept for 24 h everyday with dressing for two weeks.	[14,18,29]
29	Fungal infections	Leaves	Dry deciduous forest areas of Paschim Medinipur district, West Bengal	Paste of leaves applied locally.	[30]
30	Gum’s problems	Stems	Dhule district/Maharashtra	Brush with stem piece can also be used.	[16]
31	Headache	Roots and leaves	Ayurveda	Paste of leaves were applied.	[23]
32	Joint pain	Leaves	Tribal of Wardha district of Maharashtra	Decoction and juice	[5]
33	Liver ailments	Leaves and seeds		Paste of seeds	[31]
34	Liver diseases/jaundice	Seeds	Gujjar tribes/Sub-Himalayan region, Uttarakhand	Paste of soaked seeds was mixed with powder of *Amomum subulatum* and given with milk three times a day.	[20]
35	Manufacturing of cosmetics, wines and dyes	Stem bark, leaves and fruits	Tropical Asia and Australian tropics, Krishnagiri District, Tamil Nadu, India	Unknown	[2,8,32]
36	Ornamental pot herb	Whole plant	Tropical Asia and Australian tropics, Krishnagiri District, Tamil Nadu, India	Grown in earthen pots	[2,8,32]
37	Ringworm infections	Powdered kernel	Pune (Maharashtra)	Powdered kernel was mixed with oil and is applied topically to the affected area as a remedy for ringworm infections.	[2,23]
38	Scabies	Leaves	South West Bengal	Decoction and juice	[23]
39	Skin diseases	Leaves	Tharu community/Uttarakhand	The leaves were grounded and its paste is applied topically on affected areas.	[21,33]
40	Stomach diseases	Stem bark, leaves and fruits	Tropical Asia and Australian tropics/Krishnagiri District, Tamil Nadu, India	Decoction of leaves and barks.	[5]
41	Syphilis	Bark and roots	Ayurveda	Decoction of bark and roots.	[5,34]
42	Throat infections	Bark	Tropical Asia and Australian tropics	Bark is used internally and as a gargle in throat infections.	[23]
43	Toothache	Young branches	Pawra Tribe of Satpura Hills.	Young branches are used as toothbrushes.	[5,35]
44	Malaria and fever	Leaves	Tribe of Andaman & Nicobar Islands	Decoction of leaves	[36]
45	Ulcers	Stem	Dhule district/Maharashtra	Paste of leaves	[16]
46	Urinary tract infections	Bark and roots		Decoction of bark and roots	[5]
47	Venereal diseases	Bark and roots	Ayurveda	Decoction of bark and roots have been recommended.	[5,11,34,37]

**Table 2:** Composition of minerals from different parts of *E. laevis*.

S. No.	Mineral (g/Kg)	Leaf	Bark	Fruit
1	Sodium	2.12	1.59	1.09
2	Phosphorous	7.36	4.12	3.45
3	Calcium	38.31	38.03	36.12
4	Zinc	0.18	3.16	0.21
5	Potassium	26.23	22.33	28.12
6	Iron	1.11	0.30	1.28
7	Magnesium	12.21	6.46	13.45
8	Copper	0.02	0.05	0.01
9	Manganese	0.03	0.02	0.04
10	Silica	3.12	5.21	1.19

## Data Availability

No new data were created or analyzed in this study. Data sharing is not applicable to this article.

## Supplementary Materials

The following are available online, Table S1: Common names of *E. laevis*. [2,16,21,23,28,227,234,235].
