# Supplementary material for: Phytochemical and Ethnopharmacological Perspectives of Ehretia laevis
Source: Molecules. 2021 Jun 8;26(12):3489. doi: 10.3390/molecules26123489 (PMC8228998; doi:10.3390/molecules26123489)
Supplement: Supplementary file 1 [file molecules-26-03489-s001.zip › molecules-1226130-supplementary.pdf]

molecules

MDPI

Supplementary Materials

Phytochemical and Ethnopharmacological Perspectives of *Ehretia laevis*

Pooja Sharma [1,2], Richa Shri [1], Fidele Ntie-Kang [3,4,*] and Suresh Kumar[1,*]

[1] Department of Pharmaceutical Sciences and Drug Research, Punjabi University, Patiala-147002, Punjab, India. poojasharmagndu@gmail.com; pooja.sharma2007@yahoo.co.in (P.S.)
[2] Sri Sai College of Pharmacy, Manawala, Amritsar 143001, India.
[3] Department of Chemistry, Faculty of Science, University of Buea, P.O. Box 63 Buea, Cameroon.
[4] Institute for Pharmacy, Martin-Luther-Universität Halle-Wittenberg, Kurt-Mothes-Str. 3, 06120 Halle (Saale), Germany.
* Correspondence: fidele.ntie-kang@ubuea.cm (F.N.-K.); thakur_pu@yahoo.com (S.K.)

Table S1. Common names of *E. laevis*.

Region/Language/System of Medicine	Name
Andhra Pradesh	Paladantham
Ayurveda	Chamorar, Kapura, Pigakina, Tellapisuni, Pedd pikka, Buri, Tamboliya, Pogadi, Jhadocodkar and Tella
Gujarati	Ivory wood, vadhavaradi
Hindi	Chamar, Chamorar, Chamror, Dandos, Darar, Datranga, Khoda, Kolma, Luni, Papri, Tamboli, Tamboliya and Vadhavaradi
Jammu/Dogri	Chamror
Kanaada	Adak, Adaka, Adake, Adike, Bogari, Avak, Bondula, Haalippe, Haatippe, Halippe, Jhadocodkar, Kappura, Kapura, Karadake, Karatuke, Pigakina, Adiki, Ennebudige, Kappure and Karadaka
Konkani	Kalo gamdo
Madhyapradesh	Datrangi
Maharashtra	Ajaan, Kuptaa, Datrang, Ajaanvruksha, Khandu Chakkaand Lokhandi
Malayalam	Chavandi, Cavanti, Pedda, Sondari, Kalo gamdo, and Charandi
Marathi	Datranga, Tamboli, Kalo-Gomdo and Caranti
Nepali	Dalingal
Oriya	Mosonea
Pune	Ajan, Vruksha
Punjabi	Chamror, Konkani
Rajasthan	Tamboliya
Sanskrit	Carmivrksa, Carmakaravata, Carmavrksa, Carmiloha and Carmakasa
Siddha	Hallippe, Carmakasa, Karadaka, Papri, Kalvirusus, Pagakina and Narivalli
Tamil	Kalivirusu, Kalvirasu, Kuruviccai, Kuruvicci, Pattaiviccu, Tavittan, Narivalli, Pattaiviracu, Kalvirusus and Kuruviracu
Telugu	Aadabukkudi, Adabukkudu, Bodidhi, Bokkadi, Buri, Gidiguri, Alabukkudu, Dantam, Chilla Poku, Bukkedi

	Paladantam, Giduguri, Paladantamu, Paldatam, Paaladanthamu, Pedda pikka, Pulimaera, Peddaburimaeli, Pedda Peddapulimera, Peddapiccika, Pogadi, Pogada, Pogadichettu, Poka, Pokachettu, Polimiri, Potubokkada, Reddapulmera, Seregada, Shiragadam, Siragadam, Tella Pisini, Telladzuvvi, Tellajuvvi, Potubokada, Potubukkedi, Seregad, Shiragadamu, Siragadamu, Tellajoovi, Tellapisuni,Thellajihvi, Thellapisuni
Uttarakhand	Chamrod
West Bengal	Vadhvarni

References

2. Joshi, S.G. *Medicinal Plants*; Oxford and IBH Publishing Co. Private Ltd.: New Delhi, India, 2000, p. 102.
16. Patil, S.L.; Patil, D.A. Ethnomedicinal plants of Dhule district of Maharashtra. *Nat. Prod. Radiance* **2007**, *6*, 148–151.
21. Sharma, J.; Gairola, S.; Sharma, Y.P.; Gaur, R.D. Ethnomedicinal plants used to treat skin diseases by Tharu community of district Udham Singh Nagar, Uttarakhand, India. *J. Ethnopharmacol*. **2014**, *158*, 140–206.
23. Torane, R.C.; Kamble, G.S.; Gadkari, T.V.; Tambe, A.S.; Deshpande, N.R. GC-MS study of nutritious leaves of *Ehretia laevis*. *Int. J. Chemtech. Res.* **2011**, *3*, 1589–1591.
28. Soni, V.; Prakash, A.; Nema, M. Study on ethno medicinal botany of some plants of Dindori district of Madhya Pradesh, India. *Int. J. Pharm. Pharm. Sci.* **2012**, *8*, 1926–1929.
229. Deshpande, R.; Patil, V.; Shah, H.; Ruikar, A.; Gaikwad, S.; Kamble, G.; Mundhe, K.; Adsul, V. Back to mother nature: Novel herbal medicines in preventing dental caries. *J. Dent.* **2018**, *113*, 295–311.
236. Gaikwad, K.N.; Mali, M.V. Tree flora of Nashik city, Maharashtra. *Int. J. Life Sci. Pharm. Res.* **2012**, *2*, 94–101.
237. Rohini, I.; Jirouthu, B.; Kumar, N.S.; Bhavya, C.; Kumar, K.S. An ethno-medicinal survey of medicinal plants used by traditional healers of Araku valley, Andhra Pradesh, India. *Int. J. Curr. Res.* **2017**, *9*, 48633–48645
